# Photocurable Hydrogel Substrate—Better Potential Substitute on Bone-Marrow-Derived Dendritic Cells Culturing

**DOI:** 10.3390/ma15093322

**Published:** 2022-05-05

**Authors:** Jiewen Deng, Yao Xie, Jian Shen, Qing Gao, Jing He, Hong Ma, Yongli Ji, Yong He, Meixiang Xiang

**Affiliations:** 1 Department of Cardiology, The Second Affiliated Hospital, Zhejiang University School of Medicine, 88 Jiefang Road, Hangzhou 310009, China; dengjiewen@zju.edu.cn (J.D.); xieyao@zju.edu.cn (Y.X.); shenjian27753@zju.edu.cn (J.S.); hong_ma@zju.edu.cn (H.M.); jiyongli@zju.edu.cn (Y.J.); 2 Engineering for Life Group (EFL), Suzhou 215000, China; gaoqingvc@zju.edu.cn; 3 State Key Laboratory of Fluid Power and Mechatronic Systems, School of Mechanical Engineering, Zhejiang University, Hangzhou 310027, China; 21718670@zju.edu.cn; 4 Key Laboratory of 3D Printing Process and Equipment of Zhejiang Province, School of Mechanical Engineering, Zhejiang University, Hangzhou 310027, China

**Keywords:** GelMA-30, a novel BMDCs culturing with hydrogel substrate (CCHS), DCs function

## Abstract

Dendritic cells (DCs) are recognized as the most effective antigen-presenting cells at present. DCs have corresponding therapeutic effects in tumor immunity, transplantation immunity, infection inflammation and cardiovascular diseases, and the activation of T cells is dependent on DCs. However, normal bone-marrow-derived Dendritic cells (BMDCs) cultured on conventional culture plates are easy to be activated during culturing, and it is difficult to imitate the internal immune function. Here, we reported a novel BMDCs culturing with hydrogel substrate (CCHS), where we synthesized low substituted Gelatin Methacrylate-30 (GelMA-30) hydrogels and used them as a substitute for conventional culture plates in the culture and induction of BMDCs in vitro. The results showed that 5% GelMA-30 substrate was the best culture condition for BMDCs culturing. The low level of costimulatory molecules and the level of development-related transcription factors of BMDCs by CCHS were closer to that of spleen DCs and were capable of better promoting T cell activation and exerting an immune effect. CCHS was helpful to study the transformation of DCs from initial state to activated state, which contributes to the development of DC-T cell immunotherapy.

## 1. Introduction

Dendritic cells (DCs), known as “natural adjuvants”, have been recognized as the most effective antigen-presenting and natural vectors of antigen transmission cells in vivo. Studies have found that BMDCs reinfusion has a therapeutic effect in MI (myocardial infarction) [1], murine polymicrobial sepsis [2] and Sjögren’s-like disease [3] in mice. In recent decades, the development of DC vaccines as newly developed and promising anticancer therapies has been carried out with clinical trials of antigen-pulsed DCs for various kinds of tumors, including lung cancer [4], metastatic nasopharyngeal carcinoma [5], metastatic breast cancer [6], hepatocellular carcinoma [7], and prostate cancer [8]. Meanwhile, despite the advantages of DC vaccines, work is still required to identify their clinical efficacy [9]. Genetic engineering is a promising approach for DC vaccine development, and ectogenic genetic manipulations for embedding these tumor-associated antigens with MHC protein could further increase their immunogenicity to strengthen CD4^+^ and/or CD8^+^ cytotoxic T cell responses. In short, DCs are of importance in the mechanism study of diseases and medicine application. Research on primary cells (such as BMDCs) focuses more on internal cells’ function than cell lines. However, BMDCs induced and grown on the surface of plastic Petri dishes not only are difficult to transfect [10] and easy to be activated during culturing, but also show differences in their biological characteristics relative to DCs in the internal environment. Thus, it makes significant sense to find a substitute for plastic Petri dishes and explore how to better induce and culture BMDCs in vitro.

BMDCs are easy to be activated during culture on plastic Petri dishes; perhaps the biomaterials have a potential application to solve the dilemma. The biomaterials, which have undergone rapid development, need to demonstrate good bioactivity and formability to achieve a similar intracorporeal extracellular matrix environment and facilitate further cell development. There are some studies about the effect of polysaccharide-based hydrogels on the response of antigen-presenting cell lines [11] and strategies to reduce dendritic cell activation through PEG hydrogels loaded with immunomodulators (TGF-β1 and IL-10) during co-incubation [12]. Christine et al. evaluated the ability of a series of engineered (manufactured/fabricated) and natural collagen matrices as different treatments to either activate BMDCs or conversely induce BMDCs apoptosis in vitro [13]. In short, different biomaterials have different influences on the function of DCs [14], and this study designs and synthesizes novel biomaterials with the purpose of finding the optimal condition for BMDCs culturing.

Used as a base material of its inherently biocompatibility [15], gelatin methacrylate (GelMA, EFL-GM Series) [16] is a chemically modified gelatin, which is a denatured form of collagen with good formability and mechanical properties. It has shown promise in various areas of tissue engineering including cardiac [17,18], bone [19,20], and vascularization [21,22], as well as the development of tumor microenvironment models [23]. Meanwhile, GelMA is evidently compatible with a range of cell types, although one study showed that the GelMA culture condition drives human mononuclear cells to suppress tumor necrosis factor-a (TNF-a) expression [24]. However, there is a current lack of information regarding its interactions with immune cells, especially the effect of by GelMA on BMDCs culturing in mice in vitro.

Chakraborty et al. showed that environmental stiffness mediated by polydimethylsiloxane (PDMS) hydrogel-coated plates promotes DCs activation [25]. Thus, the characteristic of BMDCs’ easy activation during culturing was partly attributed to plastic Petri dishes with non-adjustable and stiffness substrate. There should be a better way to induce and culture BMDCs in vitro and make them more similar to the physiological microenvironment and reduce cell activation during culturing. So, we synthesized low substituted GelMA-30 hydrogels with a stiffness-adjustable characteristic and applied it to the BMDCs culture, in which a low-replacement-rate GelMA hydrogel was obtained through controlling the grafting reaction of MA with amine and hydroxy functionalities in gelatin (grafting rate is 30%). With low polymerization substitution, GelMA-30 (EFL-GM-30) is a pliant substrate suitable for cell growth, compared to highly polymerized GelMA hydrogels. In this study, firstly, 3%, 5%, 7.5%, and 10% (*w*/*v*) GelMA-30 were prepared by dissolving GelMA-30 (EFL-GM-30) in phosphate buffer saline (PBS) containing 0.25% (*w*/*v*) lithium phenyl-2,4,6-trimethylbenzoylphosphinate (LAP) at 60 °C for 30 min. Additionally, different concentrations of GelMA-30 represented different stiffness. Secondly, the different concentrations of GelMA-30 solution heated by 37 °C were immediately injected into a 24-hole plate (300 μL/hole) and then irradiated with a 405 nm light source, 3 cm, with 30 s for gelation. Then, the BMDCs suspension (5 × 10^5^ cells/hole, 400 μL) was laid on the solidified gel to form the BMDCs culturing with hydrogel substrate (CCHS). From the third day, we added fresh culture medium every day until the seventh day. At last, we aimed to find the effect of BMDCs by CCHS in vitro and whether they have a similar phenotype and function as splenic DCs in vivo.

We studied the effect and potential of hydrogel as a culturing substrate on the growth and development of BMDCs in vitro. Thus, in this study, we wanted to explore it on GelMA-30. In this study, we found that the phenotype and function of BMDCs induced and cultured in vitro on 5% GelMA-30 approached those of spleen DCs in vivo more than BMDCs cultured under normal conditions. BMDCs cultured with 5% GelMA-30 also had a strong T cell stimulation ability after LPS activation. Therefore, BMDCs culturing with hydrogel substrate (CCHS) is more conducive to studying the biological properties and immune response mechanism of DCs in vitro and contributing to the development of DC-T cell immunotherapy.

## 2. Materials and Methods

B6.Cg-Tg (TcraTcrb) 425Cbn (mutant in ovalbumin peptide residues 323–339) OT2 mice were purchased from The Jackson Laboratory (Bar Harbor, ME, USA). C57BL/6 (H-2Kb) mice were purchased from Shanghai Bing Kai experimental animal Co., Ltd. (Shanghai, China). Lithium phenyl-2,4,6-trimethylbenzoylphosphinate (LAP) was obtained from Huaxia Siyin Biotechnology Co., (Beijing, China). Monoclonal antibodies and reagents lipopolysaccharide (LPS) were purchased from Sigma (St. Luis, MO, USA). Anti-CD4 magnetic beads, anti-CD11c magnetic beads were purchased from Miltenyi Biotec (Bergisch Gladbach, Germany). CD11c, CD86, CD40, CD80, Iab, CD4, CD25, CD69, dextran-FITC and the same cognate antibody were all obtained from BD PharMingen Company (San Diego, CA, USA). RmGM-CSF, rmIL-4 were purchased from PerproTech (London, UK). Mouse TNF-a ELISA MAX™ Deluxe and Mouse IL-6 ELISA MAX™ Deluxe were obtained from Biolegend (San Diego, CA, USA).

### 2.1. Fabrication of GelMA Hydrogels

First, 10 g of gelatin was dissolved in 100 mL of DI and heated at 50 °C with stirring. Then, 0.25 mL of MA and 1% NaOH were slowly added to the above solution at the same time, and the reaction was stirred for 2 h at 50 °C in the dark. Finally, the solution was transferred to a dialysis bag (cut-off molecular weight 10 kDa), dialyzed with deionized water at 40–50 °C for 3 days and then freeze-dried. The samples were stored at 20 °C for later use.

### 2.2. Fabrication of Hydrogel Solutions

First, 0.1 g LAP was dissolved in 40 mL PBS to prepare a 0.25% (*w*/*v*) LAP at 60 °C for 30 min; secondly 3%, 5%, 7.5%, and 10% (*w*/*v*) GelMA-30 were prepared by dissolving 0.3 g, 0.5 g, 0.75 g, and 1 g hydrogel in PBS containing 0.25% (*w*/*v*) LAP buffer separately at 60 °C for 30 min. Thirdly, the different concentration GelMA-30 solutions were filtered by the 0.22 um filtering screen to obtain the sterile liquid GelMA-30 solutions and then the liquid solution was immediately injected into the 24-hole plate (300 μL/hole), then irradiated with a 405 nm light source, 3 cm, for 30 s for gelation. At last, the cell suspension (5 × 10^5^ cells/hole, 400 μL) in RPMI1640 containing 10% FBS, 10 ng/mL GM-CSF and 1 ng/mL IL-4 was laid on the solidified GelMA-30 solutions to form a two-dimensional cell culture mode. After 7 days of culture, the GelMA-30 was dissolved with a special lysate, and the cells were collected.

### 2.3. Mechanical Testing

The mechanical properties of the GelMA-30 were measured by a compressive tester (UTM-2203, Suzhou yanuotianxia Instrument Co., Ltd., Suzhou, China). To obtain a stress–strain curve, a displacement of 1 mms^−1^ was applied to the photo-crosslinked cylindrical samples with a diameter of 9 mm and a height of 6 mm. The load and displacement data by normalizing to sample cross-sectional area and height formed the stress–strain curve. The ultimate strength and failure strain were obtained from the data of broken state, and we determined the compressive modulus by the slope of the linear region of the stress–strain curve.

### 2.4. Routine Culture of Mouse Bone-Marrow-Derived Dendritic Cells (BMDCs) on GelMA-30 and Cell Morphology Observation

C57BL/6 mice were killed by anesthesia and soaked in 75% alcohol for 5 min. Bone marrow cells were washed out with RPMI1640 solution. Red blood cells were lysed with tris NH4Cl (1 mL for one mouse). After 2 min, RPMI1640 solution was added to terminate the red blood breaking process, and the filter screens were used to filter the rest tissue mass. After RPMI1640 solution washing twice, the cells were resuspended with complete medium containing 10 ng/mL GM-CSF and 1 ng/mL IL-4. The culture medium containing cells was evenly spread in a conventional 24-well plate and a 24-well plate covered with 5% GelMA-30, 0.4 mL per well, recorded as day 0. After 72 h of culture (i.e., the third day), they were rehydrated every day and cultured for 7 days, and cell morphology was observed by microscope. All studies were performed in compliance with the guideline of the Institutional Animal Care and use Committee at Zhejiang University College of Medicine.

### 2.5. Isolation and Culture of T Cells from Mouse Spleen

OT2 mice or C57 mice were killed by anesthesia and soaked in 75% alcohol for 5 min. We separated the spleen and placed it in a RPMI1640 solution, ground it, and added 1 mL Tris-NH4Cl for the red blood cell rupture. Then, we added RPMI1640 solution to terminate the reaction after 2 min and the filter screens were used to filter the rest tissue mass. The spleen cells were resuspended with MACS buffer solution (fresh RPMI1640 solution containing 2% FBS and 2 mm EDTA) for subsequent experiments. All studies were performed in compliance with guidelines of the Institutional Animal Care and Use Committee at Zhejiang University College of Medicine.

### 2.6. MACS Magnetic Beads Separate BMDCs and T Cells

The collected spleen cells or BMDCs were counted and centrifuged for 5 min (rotating speed 300× *g*) to remove the supernatant. The cells were resuspended with fresh Macs buffer containing CD4 or CD11c beads and incubated at 4 °C for at least 15 min after mixing. Using the column from the magnet, we obtained the purified cells, and we resuspended the cells with culture medium for subsequent culture and flow analysis.

### 2.7. DCs Activation Test In Vitro

The inoculated cells on the 24-well plates were stimulated by LPS in a cell incubator containing 5% CO_2_ at 37 °C. After 24 h, the supernatant of the corresponding cell group was collected and centrifuged, and the secretion of IL-6 and TNF-a in the supernatant was detected by ELISA. A comparison was then made. The cells obtained by centrifugation were used for flow cytometry and T-proliferation experiment.

### 2.8. The Secretion of Cytokines Was Detected by ELISA

The supernatant sample was taken and the specific operation was carried out according to the instructions of the ELISA kit (BioLegend, San Diego, CA, USA).

### 2.9. The Phenotype of BMDCs Was Detected by FACS

The collected BMDCs of each group were resuspended with cold 100–200 μL PBS, and then we added 0.2 μL CD11c-BV421, 0.15 μL Iab-FITC or 0.15 μL CD80-FITC, 0.2 μL CD40-APC, and 0.15 μL CD86-PE to each tube, incubating flow cytometry antibody at 4 °C for 20 min, added cold PBS to wash away excess flow cytometry antibody, centrifuging to remove supernatant, and then added cold PBS to make up 200 μL. BMDCs’ phenotypes were detected by flow cytometry.

### 2.10. T Cell Proliferation Test

CD4^+^ T cells were labeled with CFSE to form T cell suspension; then, ova323-339 peptide was added (the final concentration of ova323-339 peptide was 200 nM after co-culture with DCs), and DCs of each group were inoculated after 24 h stimulation for co-culturation (DC:T = 1:10). After co-culture for 24–48 h, 100 μL fresh medium was supplemented each hole in the 96-well plate. After co-culturation for 64 h, the cells in the well were collected, labeled with flow cytometry antibodies CD4-APC, 7AAD, CD25-PE, and CD69-PEcy7, and loaded with flow cytometry. The degree of T cell proliferation was reflected by detecting the dilution of CFSE.

### 2.11. Real-Time PCR

Total RNA was extracted from cells or using the Trizol reagent (15596018, Invitrogen, Carlsbad, CA, USA) according to the manufacturer’s instructions. In short, 500 ng of total RNA was used to make cDNA by using PrimeScript RT reagent Kit (RR037A, Takara, Shiga, Japan) according to the manufacturer’s instructions. The resulting cDNA was subjected to real-time PCR using SYBR Premix Ex Taq II (Tli RNaseH Plus) kits (RR820A, Takara, Shiga, Japan) on an Applied Biosystems Q7 Fast Real-Time PCR System (ABI, Torrance, CA, USA). PCR was performed with the following conditions: denaturation temperature 95 °C for 0.5 min, annealing temperature (according to respective primer) for 0.5 min, and extension temperature 72 °C for 1 min, and the PCR cycle was determined according to a kinetic profile. β-actin was used as an internal loading control to normalize all PCR products. All primers information is as follows:
IRF4Forward PrimerCTTTGAGGAATTGGTCGAGAGG
Reverse PrimerGAGAGCCATAAGGTGCTGTCAIRF8Forward PrimerAGACCATGTTCCGTATCCCCT
Reverse PrimerCACAGCGTAACCTCGTCTTCCBatf3Forward PrimerCAGAGCCCCAAGGACGATG
Reverse PrimerGCACAAAGTTCATAGGACACAGCβ-ActinForward PrimerAGATCAAGATCATTGCTCCTCCT
Reverse PrimerACGCAGCTCAGTAACAGTCC

### 2.12. Phagocytosis Assay and Flow Cytometry

BMDCs phagocytosis was performed by treating BMDCs stimulated by LPS for 24 h together with dextran-FITC (100 μg/mL), which was divided into two groups: one group was cultured in 4 °C for 2 h, the other in 37 °C for the same time. Then, BMDCs were washed by PBS twice and were binded with CD11c monoclonal antibodies at 4 °C for 20 mins to sort out DCs by FACS. Flow cytometry analysis was performed using the application Flowjo.

### 2.13. Statistical Analysis

The GraphPad Prism 6.0 analysis software was used for statistical analysis. The unpaired sample *t* test was used for the comparison between the two samples, and one-way ANOVA was used for the mean comparison between multiple groups. The measurement data were expressed in mean ± SD. The difference was statistically significant (*p* < 0.05).

## 3. Result

### 3.1. BMDCs on GelMA-30 Substrates

**Figure 1:**
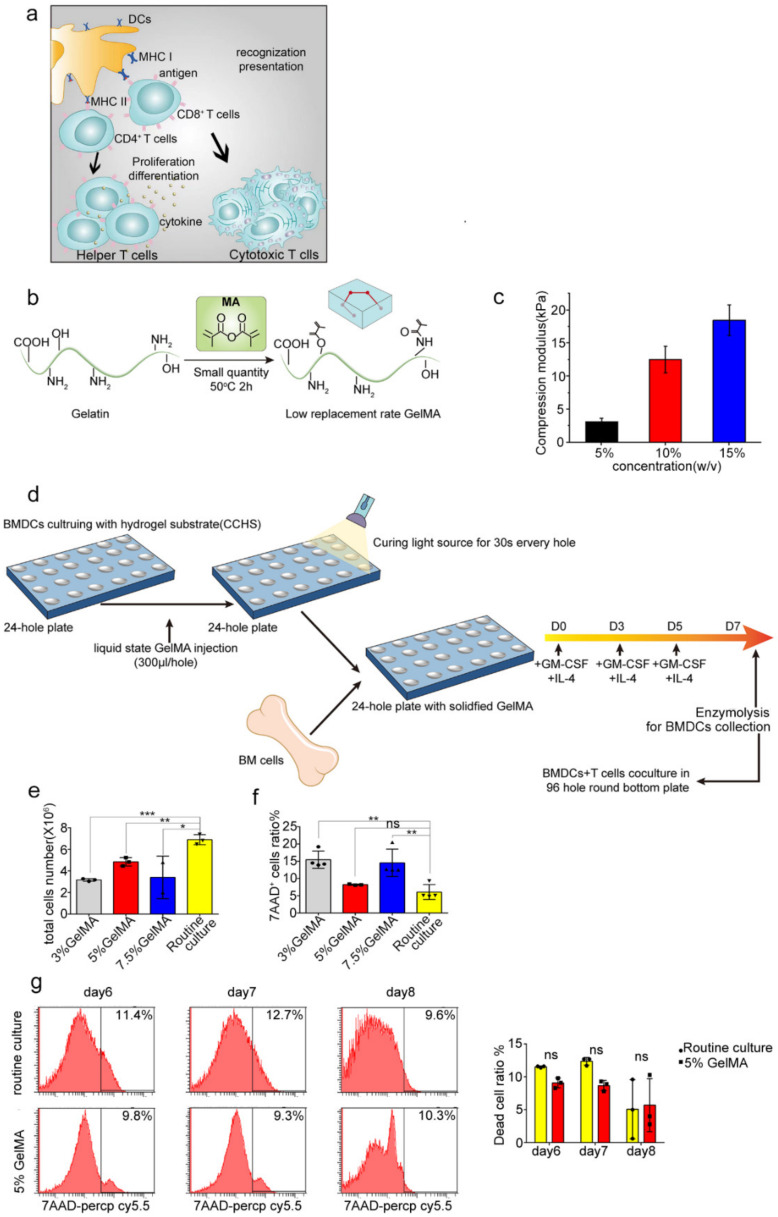
BMDCs on GelMA-30 substrates. (**a**) The interaction between DCs and T cells. (**b**) Synthetic equation of GelMA-30 hydrogels with low substitution rate. (**c**) The table about the Compression modulus of GelMA-30 with different concentrations. (**d**) The progress of BMDCs by CCHS: BMDCs were cultured with GM-CSF (10 ng/uL) and IL-4 (1 ng/uL) from C57 mice on stiffness adjustable hydrogel substrate GelMA-30. (**e**–**g**) BMDCs by CCHS or on normal condition, respectively, were collected, respectively, and bound with 7AAD-percpcy5.5, with the observation and comparison by FACS. These experiments were repeated twice with essentially the same results. In all experiments, each picture was collected as means ± SEM, data were analyzed with unpaired student’s *t* test or analysis variance (ANOVA) and multiple comparison, and * *p* < 0.05, ** *p* < 0.01, *** *p* < 0.001, ns: nonsignificant.

DCs play significant role in priming T cells responses, as Figure 1a depicts. In order to better culture BMDCs and solve the problem of easy activation, partly owing to the stiffness during BMDCs culturing, we synthesized the low substituted GelMA-30 hydrogels with stiffness adjustable and applied it to the BMDCs culture. Figure 1b demonstrates the simple and general synthesis steps of GelMA-30. It can be clearly seen that a low replacement rate GelMA hydrogel was obtained through controlling the grafting reaction of MA with amine and hydroxy functionalities in gelatin (grafting rate is 30%). Because of the low polymerization substitution, GelMA-30 (EFL-GM-30) has a pliant substrate suitable for cell growth, compared to the highly polymerized GelMA hydrogels. Furthermore, different concentrations of GelMA-30 (EFL-GM-30) represented different stiffness. As is shown in Figure 1c, the elastic modulus of GelMA-30 under compression at fully swollen state varies significantly with GelMA-30 concentration, and the higher the concentration of GelMA-30, the greater the mechanical stiffness. According to the characteristics of semisuspension and semiadherence of BMDCs, we then used GelMA-30 with different concentrations as a substitute for the conventional culture plates on the culture and induction of BMDCs in vitro (Figure 1d), which was named the BMDCs culturing with hydrogel substrate (CCHS). To find the suitable stiffness for BMDCs culturing, 10% GelMA-30 was first applied to culture BMDCs. A total of 78.2% of cells died after 6 days of culture (Figure S1), indicating that 10% GelMA-30 was not usable for BMDCs culture because of its stiffness with the higher polymerization amount in the substrate. Then, we tried to culture BMDCs on 3%, 5%, and 7.5% GelMA-30. After several days of culture, it was found that the cells cultured on the 5% GelMA-30 could survive normally based on observations demonstrating no significant difference, with a higher total cell number compared to the 3%, 7.5% GelMA-30 group (Figure 1e) and the lowest dead cells ratio (Figure 1f,g). However, the number of cells cultured in the 5% GelMA-30 group was still lower than that in the conventional culture system (Figure 1e). Therefore, cells could survive on 5% GelMA-30 with the lower dead cells ratio; 5% GelMA-30 is the appropriate concentration and stiffness for BMDCs culture.

### 3.2. BMDCs Were Induced and Cultured Successfully on 5% GelMA-30

**Figure 2:**
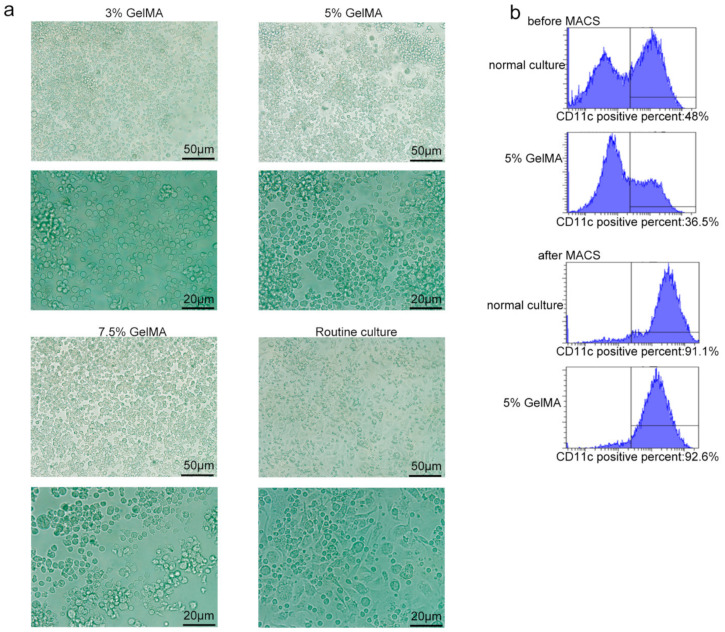
BMDCs on GelMA-30 with the morphology of fewer tentacles. (**a**) BMDCs by CCHS (3%, 5% and 7.5% GelMA-30) or on normal condition, respectively, were collected the seventh day and the morphology was observed under the microscope. (**b**) BMDCs were collected on the sixth day for the purity by MACS and be observed by FACS about the proportion of the CD11c+ cells. These experiments were repeated twice with essentially the same results.

**Figure 3:**
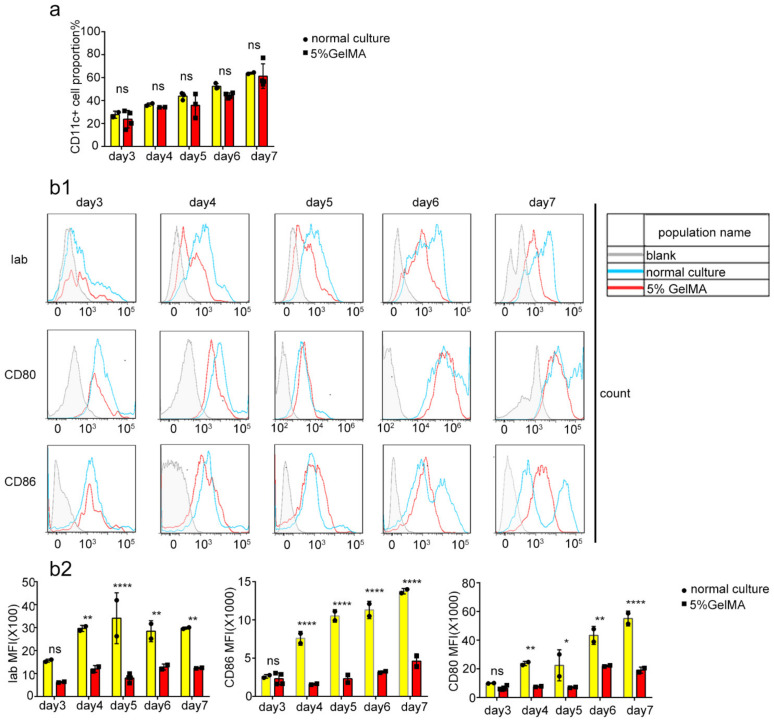
BMDCs were induced and cultured successfully on 5% GelMA-30. (**a**) BMDCs by CCHS or on normal condition, respectively, were collected (from the third to the seventh day) every day, respectively, and bound with CD11c, with the observation and comparison by FACS. (**b1**,**b2**) BMDCs by CCHS or on normal condition, respectively, were collected (from the third to the seventh day) every day, respectively, and bound with Iab, CD80, CD40, with the observation and comparison by FACS. These experiments were repeated twice with essentially the same results. In all experiments, each picture was collected as means ± SEM, data were analyzed with unpaired student’s *t* test or analysis variance (ANOVA) and multiple comparison, and * *p* < 0.05, ** *p* < 0.01, **** *p* < 0.0001, ns: nonsignificant. Mean fluorescence intensity, MFI.

Theoretically, CD11c is a specific marker of the successful induction of BMDCs, while mature DC markers express the costimulatory molecules CD80, CD86, MHC-II, etc. [26,27]. To further understand the growth status of cells on 5% GelMA-30 and the induction of BMDCs, the microscopic results showed that the cells cultured on the surface of 5% GelMA-30 grew with agglomeration, which was similar to the conventional culture group. However, 7 days post-cell culture, the rounder cells cultured on GelMA-30 produced fewer tentacles (Figure 2a and Figure S2). Additionally, the proportion of CD11c+ cells at day 7 reached more than 90% for the 5% GelMA-30 and normal culture groups with cell purification by magnetic bead sorting (Figure 2b). Additionally, the flow cytometry results revealed that the proportion of CD11c^+^ cells was similar with no significant difference (Figure 3a) between the bone marrow cells in conventional culture and the cells on 5% GelMA-30 over 3–7 days, although the immune fluorescence data revealed that expression of the markers Iab, CD80 and CD86 of CD11c^+^ cells cultured on 5% GelMA-30 was much lower than that of BMDCs by the conventional culture (Figure 3(b1,b2)), illustrating the low expression level of these costimulatory molecules of BMDCs by CCHS. Therefore, BMDCs could be successfully induced in vitro by the novel culture method, the BMDCs culturing with hydrogel substrate (CCHS).

### 3.3. BMDCs on 5% GelMA-30 with Low Expression Levels of Costimulatory Molecules Were Activated by LPS

**Figure 4:**
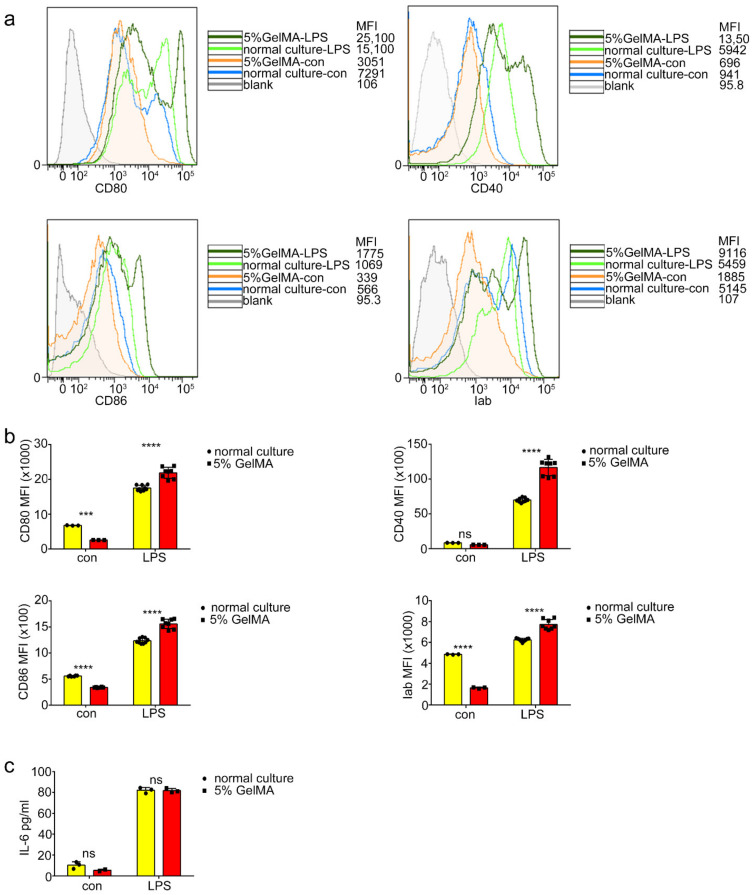
BMDCs cultured on 5% GelMA-30 with the low expression level of costimulatory molecules could be well activated by LPS as well. (**a**,**b**) BMDCs by CCHS or on normal condition, respectively, were collected, respectively, with the 24 h LPS stimulation (100 ng/mL) and bound with CD11c, Iab, CD80, CD40, with the observation and comparison by FACS. These experiments were repeated twice with essentially the same results. (**c**) Culture supernatants were collected 24 h after LPS stimulation, and cytokine (IL-6) levels were measured using ELISA. In all experiments, each picture was collected as means ± SEM, data were analyzed with unpaired student’s *t* test or analysis variance (ANOVA) and multiple comparison, and *** *p* < 0.001, **** *p* < 0.0001, ns: nonsignificant. Mean fluorescence intensity, MFI.

**Figure 5:**
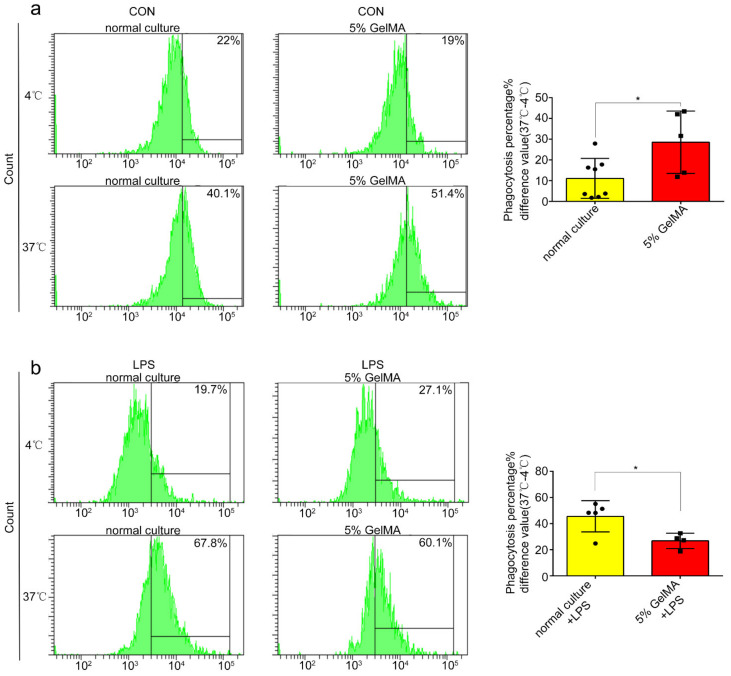
BMDCs cultured on 5% GelMA-30 with more potential for antigen uptake. (**a**,**b**) BMDCs by CCHS or on normal condition, respectively, were pretreated with 100 ng/mL LPS for 24 h and then incubated with 100 μg/mL FITC-dextran for 1 h, setting two groups—one group was cultured in 4 degrees as the negative control, another in 37 °C in favor of cells phagocytosis. Then, BMDCs were bound with CD11c-BV421 and the dextran was detected in the FITC channel in FACS. In all experiments, each picture was collected as means ± SEM, data were analyzed with unpaired student’s *t* test or analysis variance (ANOVA) and multiple comparison, and * *p* < 0.05.

To determine whether the BMDCs culturing on 5% GelMA-30 can be activated by LPS, we tested the BMDCs costimulatory molecule expression and the inflammatory cytokine secretion of BMDCs with LPS treatment. We found that the mean fluorescence intensity of the costimulatory molecule expressed (Iab, CD40, CD80 and CD86) on BMDCs by CCHS was significantly higher than that of BMDCs from the normal culture group (Figure 4a,b) after 24 h LPS stimulation, while the secretion of Interleukin-6 (IL-6) increased with no significant difference (Figure 4c) in both groups. These results demonstrated that BMDCs culturing on 5% GelMA-30 had better potential for maturation by LPS stimulation. At resting state, DCs are considered immature, with high endocytic capability and expressing low levels of MHC and costimulatory molecules [28], and dextran is recognized and taken up by macrophages, DCs, LSECs, and some other preferred cell types via specific receptors [29,30,31,32]. We found that BMDCs on 5% GelMA-30 promoted dextran phagocytosis (Figure 5a) compared to the normal culture group, whereas with 24 h LPS stimulation, the CCHS group inhibited dextran phagocytosis (Figure 5b). BMDCs by CCHS had more potential for antigen uptake and were more immature than those collected from the control group.

### 3.4. BMDCs on GelMA-30 Approached Spleen DCs

**Figure 6:**
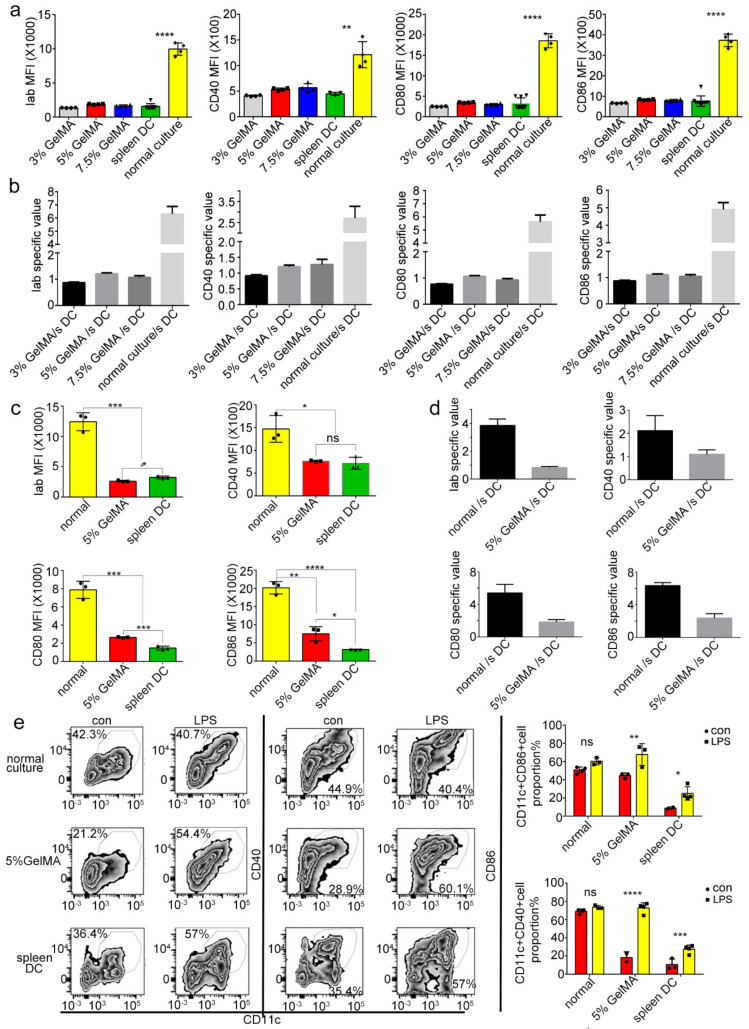
BMDCs cultured on 5% GelMA-30 were closer to the spleen DCs on phenotype and function in vivo. (**a**,**c**) BMDCs were collected from different culture systems, respectively. Then, the spleen DCs were collected by MACS to make the comparison for the expression of the costimulatory molecule with the application of FACS. (**b**,**d**) The costimulatory molecule specific values were obtained from the ratio of the MFI data of BMDCs from different culture systems, respectively, to the data of the spleen DC group. “s DC” represents the spleen DC. (**e**) BMDCs and spleen DCs were stimulated by LPS (100 ng/mL) for 24 h. Then, the cells were bound with Iab, CD40, CD80 and CD86 and were analyzed and compared in FACS. These experiments were repeated twice with essentially the same results. In all experiments, each picture was collected as means ± SEM, data were analyzed with unpaired student’s *t* test or analysis variance (ANOVA) and multiple comparison, and * *p* < 0.05, ** *p* < 0.01, *** *p* < 0.001, **** *p* < 0.0001, ns: nonsignificant. Mean fluorescence intensity, MFI.

Based on the previous results, are the low expression levels of costimulatory molecules on BMDCs by CCHS closer to the phenotype and function of spleen DCs in vivo than BMDCs by routine culture in vitro? To find the answer, we tested and compared the expression level of costimulatory molecules on spleen DCs and BMDCs from different culturing substrates. The FACS analysis results showed that the low expression levels of costimulatory molecules (Iab, CD80, CD86, CD40) on BMDCs cultured on 5% GelMA-30, as well as on the 3% and 7.5% GelMA-30, were all similar to those of spleen DCs (Figure 6a,c). Meanwhile, according to the data from the spleen DCs used as controls, the specific value of costimulatory molecules (Iab, CD80, CD86, CD40) in the 5% GelMA-30 group/spleen DCs group approached 1 (Figure 6b,d). It suggests that the low expression levels of costimulatory molecules on BMDCs cultured on 5% GelMA-30 were closer to the phenotype of spleen DCs in vivo with the evidence of the appropriate stiffness and good biocompatibility of GelMA-30 (EFL-GM-30) for BMDCs culturing. To further study the corresponding functions of BMDCs cultured on different substrates, as is shown in Figure 6e, the activated CD11c^+^ population expressing CD40 and CD86 on 5% GelMA-30 BMDCs significantly increased post-LPS stimulation, and the proportion of the activated cell population on spleen DCs also rose to a certain extent, but the proportion of the activated subpopulation of BMDCs by routine culturing did not significantly change after LPS stimulation (Figure 6e). To sum up, a larger proportion of cells were matured on BMDCs by CCHS with LPS treatment, which is beneficial for studying the transformation mechanism of DCs from initial state to activated state.

**Figure 7:**
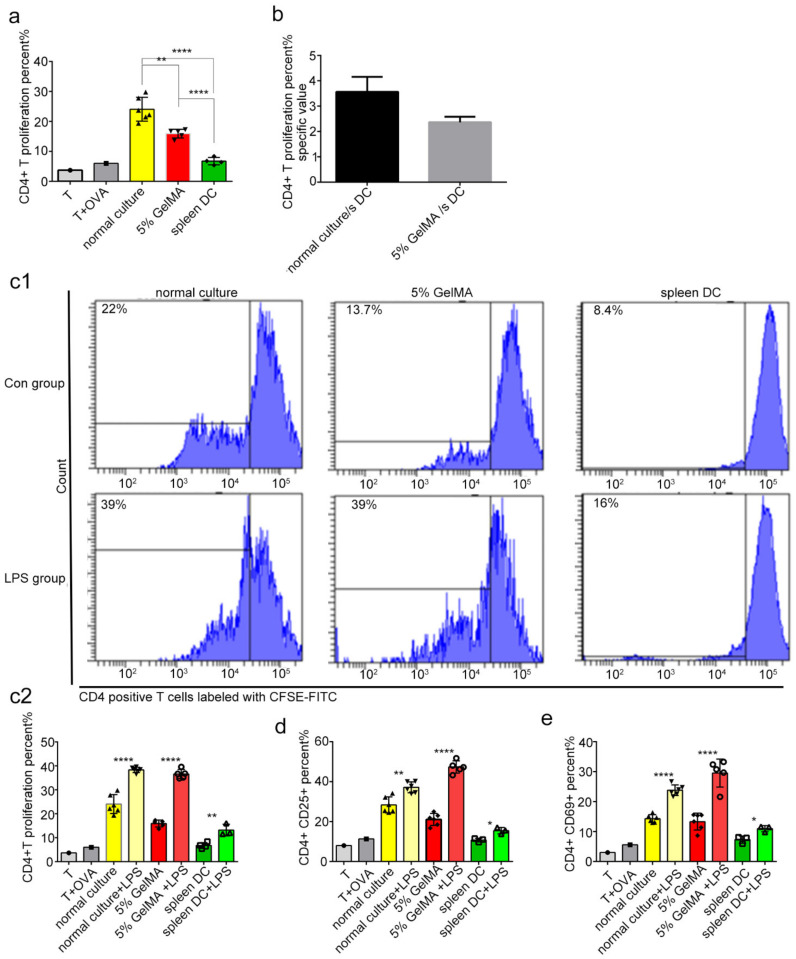
BMDCs cultured on 5% GelMA-30 were closer to the spleen DCs with more potential to activate T cells. (**a**,**c**–**e**) BMDCs from different culture systems and spleen DCs were stimulated with 100 ng/mL LPS, respectively, for 24 h. After 24 h, the collected DCs (10^4^ cells) were co-cultured with CD4^+^ T cells (10^5^ cells bound with CFSE-FITC) extracted from OT2 mice’s spleen with the existence of OVA-17 peptides for 64 h. After 64 h, the proliferation peak of CD4^+^ T cells and CD25^+^ or CD69^+^ cells were observed and measured using flow cytometry. Numbers above bracketed lines indicate percent CFSE low (proliferated) cells. Right, frequency of rapidly dividing cells among those at left. (**b**) The CD4^+^ T proliferation percent specific value was obtained from the ratio of the data of BMDCs from different culture systems, respectively, to the data of the spleen DC group. “s DC” represents the spleen DC. These experiments were repeated twice with essentially the same results. In all experiments, each picture was collected as means ± SEM, data were analyzed with unpaired student’s *t* test or analysis variance (ANOVA) and multiple comparison, and * *p* < 0.05, ** *p* < 0.01, **** *p* < 0.0001.

**Figure 8:**
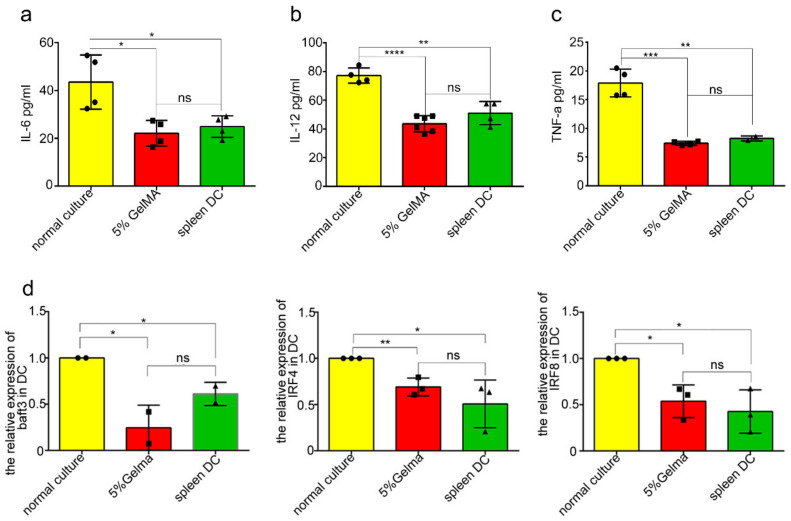
BMDCs cultured on 5% GelMA-30 were closer to the spleen DCs on developmental state. (**a**–**c**) BMDCs from different culture systems and spleen DCs cultured in the 24-hole plate. Then, culture supernatants were collected 24 h, and cytokine (IL-6, IL-12, TNF-a) levels were measured using ELISA. (**d**) The relative expression of IRF4, IRF8, baft3, and Notch2 of DCs from different culture systems and spleen DCs were measured by qPCR. Data points are represented as mean ± SD for triplicate measurements. These experiments were repeated twice with essentially the same results. In all experiments, each picture was collected as means ± SEM, data were analyzed with unpaired student’s *t* test or analysis variance (ANOVA) and multiple comparison, and * *p* < 0.05, ** *p* < 0.01, *** *p* < 0.001, **** *p* < 0.0001, ns: nonsignificant.

Theoretically, DCs were able to present antigens and contribute to the proliferation of T cells, and the increased expression of CD25 and CD69 is the marker of T cell activation [33,34]. Therefore, the BMDCs were cocultured with CD4^+^ T cells for the T proliferation experiment. In the unstimulated state, the proliferation and activation level of CD4^+^ T cells stimulated by BMDCs in the GelMA-30 group by CCHS approached that in the spleen DCs group (Figure 7a). Additionally, Figure 7b showed that the 5% GelMA-30 culture group/spleen DCs group-specific CD4^+^ T cell proliferation percentage approached 1. It illustrated that the function of BMDCs by CCHS at the resting state approached the role of spleen DCs. The activated population of costimulatory molecules (CD40^+^ and CD86^+^) in the GelMA-30 group grew most apparently after LPS stimulation, which resulted in the widest proliferation ratio change of CD4^+^ T cells (Figure 7c) and the highest proportion of activated T cell populations expressing CD25 and CD69 (Figure 7d,e). In other words, BMDCs by CCHS post-maturation by LPS were more capable of triggering T cell responses. On the other hand, BMDCs cultured on 5% GelMA-30 secreted IL-6, IL-12, and TNF-a at similar levels with no significant difference compared to the spleen DC group (Figure 8a–c). In summary, LPS stimulation can also activate BMDCs by CCHS, which contributes to the development of DC-T cell immunotherapy.

### 3.5. BMDCs on 5% GelMA-30 Were Closer to Spleen DCs in Terms of the Developmental State

Murine DCs subsets consisted of IRF8-dependent conventional (c)DC1s, IRF4-dependent cDC2s, and monocyte-derived DCs. During the differentiation and development of dendritic cells, cDC1s express a high level of the transcription levels of Interferon regulatory factor 8 (IRF8) and rely on IRF8 [35,36], basic leucine zipper ATF-like transcription factor3 (Batf3) [37,38], DNA-binding2 (ID2) [39,40], nuclear factor, interleukin 3 regulated (Nfil3) [41], and B cell lymphoma 6 (Bcl6) for development [42,43], while cDC2s express Interferon regulatory factor 4 (IRF4) and IRF8, although the expression level is lower than that of cDC1s cells. Therefore, we used qPCR to detect and analyze the transcription levels of DCs development-related factors among the spleen DCs and BMDCs by CCHS or culturing on the plastic plate. The results showed that there was no significant difference in the IRF4, IRF8, and Batf3 transcription levels of BMDCs culturing on the 5% GelMA-30 and spleen DCs, yet these transcription levels of BMDCs culturing on the plastic plate were higher (Figure 8d). Thus, we came to the conclusion that the functional phenotype and developmental state of BMDCs by CCHS were closer to those in the spleen DCs group, which further proved the appropriate stiffness and good biocompatibility of GelMA-30 (EFL-GM-30) as the better settlement of easy activation during BMDCs culture.

## 4. Discussion

Recently, with the development of 3D printing, biomaterials are more often used in medicine and cell biology. For example, a biomaterials approach and active devices contributed to the development and translation of epicardial therapies for myocardial infarction [44], and material stiffness influences the polarization state, function, and migration mode of macrophages [45]. Can novel materials (GelMA) be used for culturing cells? Because of the importance of DCs and the problem of BMDCs culture on the plastic plates, we applied the photocurable GelMA hydrogels to the BMDCs culture and observed the effect.

In this study, the lack of amino groups and the high polymerization degree on the highly polymerized GelMA hydrogels made it have poor solubility and unadjusted stiffness. Therefore, we synthesized the low substituted GelMA-30 hydrogels (EFL-GM-30), in which one amino group is substituted by polymerization with a 30% substitution rate (Figure 1b) and used it as a substitute for plastic Petri dishes on the culture of BMDCs in vitro, which was named the BMDCs culturing with hydrogel substrate (CCHS). GelMA-30 was able to dissolve in PBS at 60 °C and different concentrations of GelMA-30 had different stiffness. We found that BMDCs on 5% GelMA-30 have a lower dead cells ratio compared to the 3% and 7.5% groups, so 5% GelMA-30 was the best concentration and condition for BMDCs culture. During the culture period, the generally low expression levels of costimulatory molecules on 5% GelMA were similar to the expression levels of costimulatory excitons on spleen DCs, which proved the high biocompatibility of GelMA-30. Moreover, the BMDCs by CCHS had larger potential for antigen uptake.

DCs generally need three signals to activate the T cell immune response: the first signal is the complex formed by the binding of MHC molecules and antigen peptides, which transmits antigen information to T cell surface receptors; the second signal is the interaction between some costimulatory molecules of DCs (CD86, CD80, CD40) and T cell surface molecules (CD28); the third signal is the cytokines secreted by DCs, such as IL-12, TNF-a, and IL-10, which directly affect the direction of T cell polarization [46,47,48]. The increased expression of CD25 and CD69 is a marker of T cell activation; CD69 is one of the earliest upregulated markers after T cell activation [33], and CD25 is an IL-2 receptor with the capability of activating T lymphocytes and further producing IL-2 [34]. We found that the proliferation and activation proportion of CD4^+^ T cells after encountering the resting BMDCs by CCHS was closer to that of the spleen DCs; after LPS stimulation, BMDCs culturing on 5% GelMA triggered the most CD4^+^ T cells proliferation and activation. At the same time, the qPCR results of transcription levels of development-related factors showed that the transcription levels of IRF4, IRF8, and Batf3 in BMDCs cultured on 5% GelMA-30 were similar to those of spleen DCs. These results illustrated that the initial state of BMDCs by CCHS in vitro was closer to that of spleen DCs, which expressed low levels of costimulatory molecules and similar development-related factors. LPS stimulation can also drive BMDCs by CCHS mature, which is more conducive to the study of the transformation of DCs from the initial state to a matured state. The low substituted photocurable hydrogel substrate (GelMA-30) is convenient for finding BMDCs culturing substrate stiffness, partly because of its adjustable stiffness and good biocompatibility. Additionally, 5% GelMA-30 is the appropriate stiffness for BMDCs culture because of its lowest dead-cell ratio during culture.

The function of DCs mainly depends on their activation and maturation. Controlling the activation and maturation of DCs is of great significance in the treatment of many clinical diseases and vaccine production [49]. Moreover, the specific mechanism of DCs in the process of immune tolerance and immune activation is not clear [50]. BMDCs by CCHS with a low expression level of costimulatory molecules were also activated by LPS, which is beneficial for promoting the development of DCs vaccines. However, the culture system has the problems of a small number of cells and high cost, so the culture materials and conditions still need to be further developed and explored. Most importantly, BMDCs by CCHS were closer to spleen DCs in terms of phenotype and function in vivo, able to better promote T cell activation and exert the immune effect. CCHS is helpful to study the transformation of DCs from initial state to activated state, which contributes to the development of DC-T cell immunotherapy.

## 5. Conclusions

In this study, to solve the problem of easy activation, partly owing to the stiffness during BMDCs culturing, we synthesized GelMA-30 and demonstrated the effect of low substituted GelMA-30 hydrogels on the growth and development of BMDCs. Additionally, a novel BMDCs culturing with hydrogel substrate (CCHS) using the low substituted GelMA-30 was reported. At resting state, BMDCs culturing on the 5% GelMA-30 substrate was closer to that of spleen DCs; after LPS stimulation, they are capable of better promoting T cell activation and exerting immune effect, contributing to the development of DC-T cell immunotherapy. The function of DCs mainly depends on their activation and maturation. Controlling the activation and maturation of DCs is of great significance in the treatment of many clinical diseases and vaccine production. Moreover, the specific mechanism of DCs in the process of immune tolerance and immune activation is not clear. BMDCs by CCHS with a low expression level of costimulatory molecules were also matured by LPS, which is beneficial for promoting the development of DCs vaccines.

## Supplementary Materials

The following supporting information can be downloaded at: https://www.mdpi.com/article/10.3390/ma15093322/s1, Figure S1: GelMA-30. BMDCs were cultured with GM-CSF (10 ng/μL) and IL-4 (1 ng/μL) from C57 mice with the application of GelMA-30 by 10% GelMA-30 or on normal condition respectively. Then the BMDCs on day 6 (or day 7 and day 8) were collected respectively and bound with 7AAD-percpcy5.5, with the observation and comparison by FACS; Figure S2: The morphology of BMDCs at different culture day. BMDCs by CCHS or on normal condition respectively were collected (from the third to the seventh day) every day and the morphology was observed under the microscope. These experiments were repeated twice with essentially the same results.

## Abbreviations

CCHS	BMDCs culturing with hydrogel substrate
BMDCs	Bone marrow-derived Dendritic cells
DCs	Dendritic cells
cDCs	Conventional Dendritic cells
LPS	Lipopolysaccharide
PBS	Phosphate buffer saline
GelMA-30	Gelatin Methacrylate-30
LAP	Lithium phenyl-2,4,6-trimethylbenzoylphosphinate
GM-CSF	Granulocyte/macrophage colony stimulating factor (GM-CSF)
TNF-a	Tumor necrosis factor-α
IL	Interleukin
IRF	Interferon regulatory factor
Batf	Basic leucine zipper ATF-like transcription factor
ID2	DNA-binding2
Nfil3	Nuclear factor, interleukin 3 regulated
Bcl6	B cell lymphoma 6
