# Supplementary material for: Photocurable Hydrogel Substrate—Better Potential Substitute on Bone-Marrow-Derived Dendritic Cells Culturing
Source: Materials (Basel). 2022 May 5;15(9):3322. doi: 10.3390/ma15093322 (PMC9104740; doi:10.3390/ma15093322)
Supplement: Supplementary file 1 [file materials-15-03322-s001.zip › materials-1616076-supplementary.pdf]

Supplementary Information

Photocurable hydrogel substrate—better potential substitute on Bone-marrow-derived Dendritic cells Culturing

Jiewen Deng[1,2], Yao Xie[1,2], Jian Shen[1,2], Qing Gao[3], Jing He[4,5], Hong Ma[1,2], Yongli Ji[1,2], Yong He[4,5,*] and Meixiang Xiang[1,2,*]

[1] Department of Cardiology, The Second Affiliated Hospital, Zhejiang University School of Medicine, 88 Jiefang Road, Hangzhou 310009, China; dengjiewen@zju.edu.cn (J.D.); xieyao@zju.edu.cn (Y.X.); shenjian27753@zju.edu.cn (J.S.); hong_ma@zju.edu.cn (H.M.); jiyongli@zju.edu.cn (Y.J.)

[2] Cardiovascular Key Laboratory of Zhejiang Province, Hangzhou 310009, China[3] Engineering for Life Group (EFL), Suzhou 215000, China; gaoqingvc@zju.edu.cn

[4] State Key Laboratory of Fluid Power and Mechatronic Systems, School of Mechanical Engineering, Zhejiang University, Hangzhou 310027, China; 21718670@zju.edu.cn

[5] Key Laboratory of 3D Printing Process and Equipment of Zhejiang Province, School of Mechanical Engineering, Zhejiang University, Hangzhou 310027, China

* Correspondence: yongqin@zju.edu.cn (Y.H.); xiangmx@zju.edu.cn (M.X.)

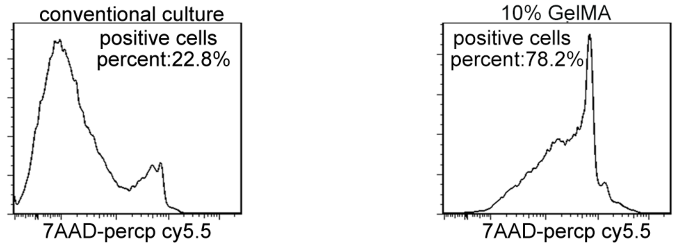

Figure S1. GelMA-30. BMDCs were cultured with GM-CSF (10ng/ul) and IL-4 (1 ng/μL) from C57 mice with the application of GelMA-30 by 10% GelMA-30 or on normal condition respectively. Then the BMDCs on day 6 (or day 7 and day 8) were collected respectively and bound with 7AAD-percpcy5.5, with the observation and comparison by FACS.

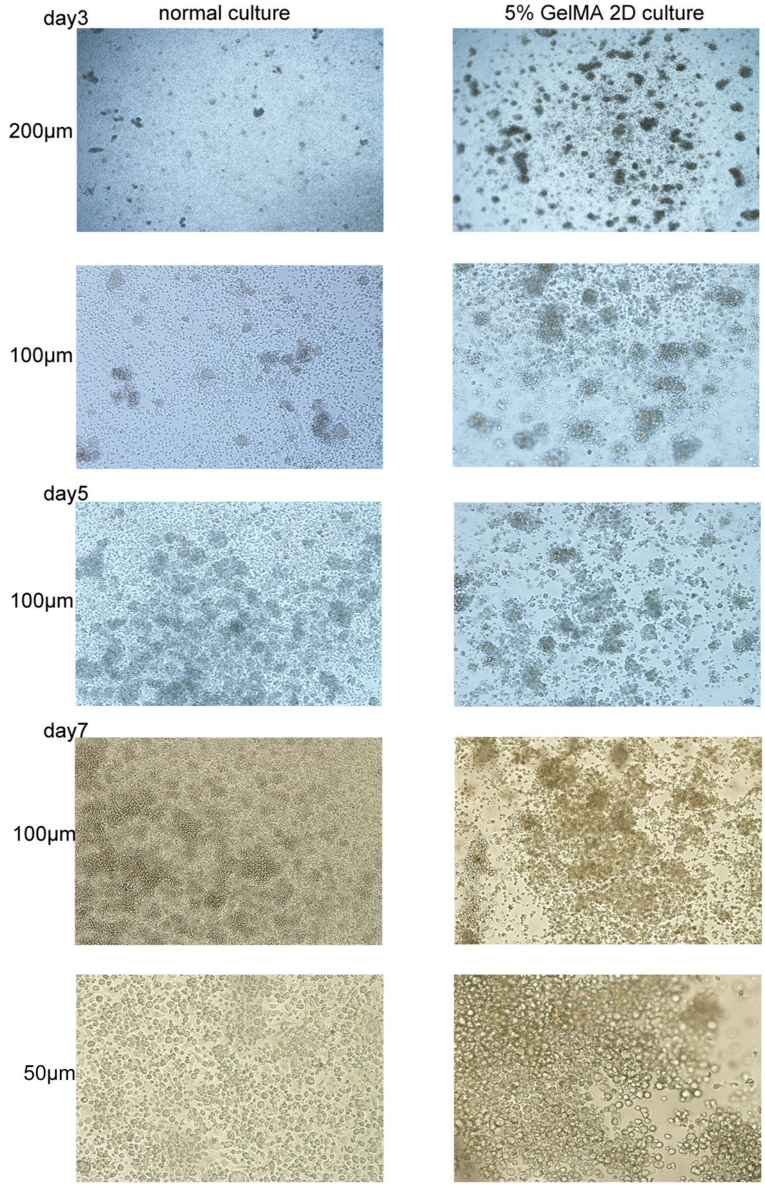

Figure S2. The morphology of BMDCs at different culture day. BMDCs by CCHS or on normal condition respectively were collected (from the third to the seventh day) every day and the morphology was observed under the mi-croscope. These experiments were repeated twice with essentially the same results.
